# Anti-Tumor Effects of Chinese Medicine Compounds by Regulating Immune Cells in Microenvironment

**DOI:** 10.3389/fonc.2021.746917

**Published:** 2021-10-14

**Authors:** Fengqian Chen, Jingquan Li, Hui Wang, Qian Ba

**Affiliations:** State Key Laboratory of Oncogenes and Related Genes, Center for Single-Cell Omics, School of Public Health, Shanghai Jiao Tong University School of Medicine, Shanghai, China

**Keywords:** Chinese medicine compounds, cancer, immune cell, antitumor immunity, microenvironment

## Abstract

As the main cause of death in the world, cancer is one of the major health threats for humans. In recent years, traditional Chinese medicine has gained great attention in oncology due to the features of multi-targets, multi-pathways, and slight side effects. Moreover, lots of traditional Chinese medicine can exert immunomodulatory effects *in vivo*. In the tumor microenvironment, tumor cells, immune cells as well as other stromal cells often coexist. With the development of cancer, tumor cells proliferate uncontrollably, metastasize aggressively, and modulate the proportion and status of immune cells to debilitate the antitumor immunity. Reversal of immunosuppressive tumor microenvironment plays an essential role in cancer prevention and therapy. Immunotherapy has become the most promising strategy for cancer therapy. Chinese medicine compounds can stimulate the activation and function of immune cells, such as promoting the maturation of dendritic cells and inducing the differentiation of myeloid-derived suppressor cells to dendritic cells and macrophages. In the present review, we summarize and discuss the effects of Chinese medicine compounds on immune cells in the tumor microenvironment, including innate immune cells (dendritic cells, natural killer cells, macrophages, and myeloid-derived suppressor cells) and adaptive immune cells (CD4^+^/CD8^+^ T lymphocytes and regulatory T cells), and the various immunomodulatory roles of Chinese medicine compounds in cancer therapy such as improving tumor-derived inflammation, enhancing the immunity after surgery or chemotherapy, blocking the immune checkpoints, et al., aiming to provide more thoughts for the anti-tumor mechanisms and applications of Chinese medicine compounds in terms of tumor immunity.

## Introduction

**Figure 1:**
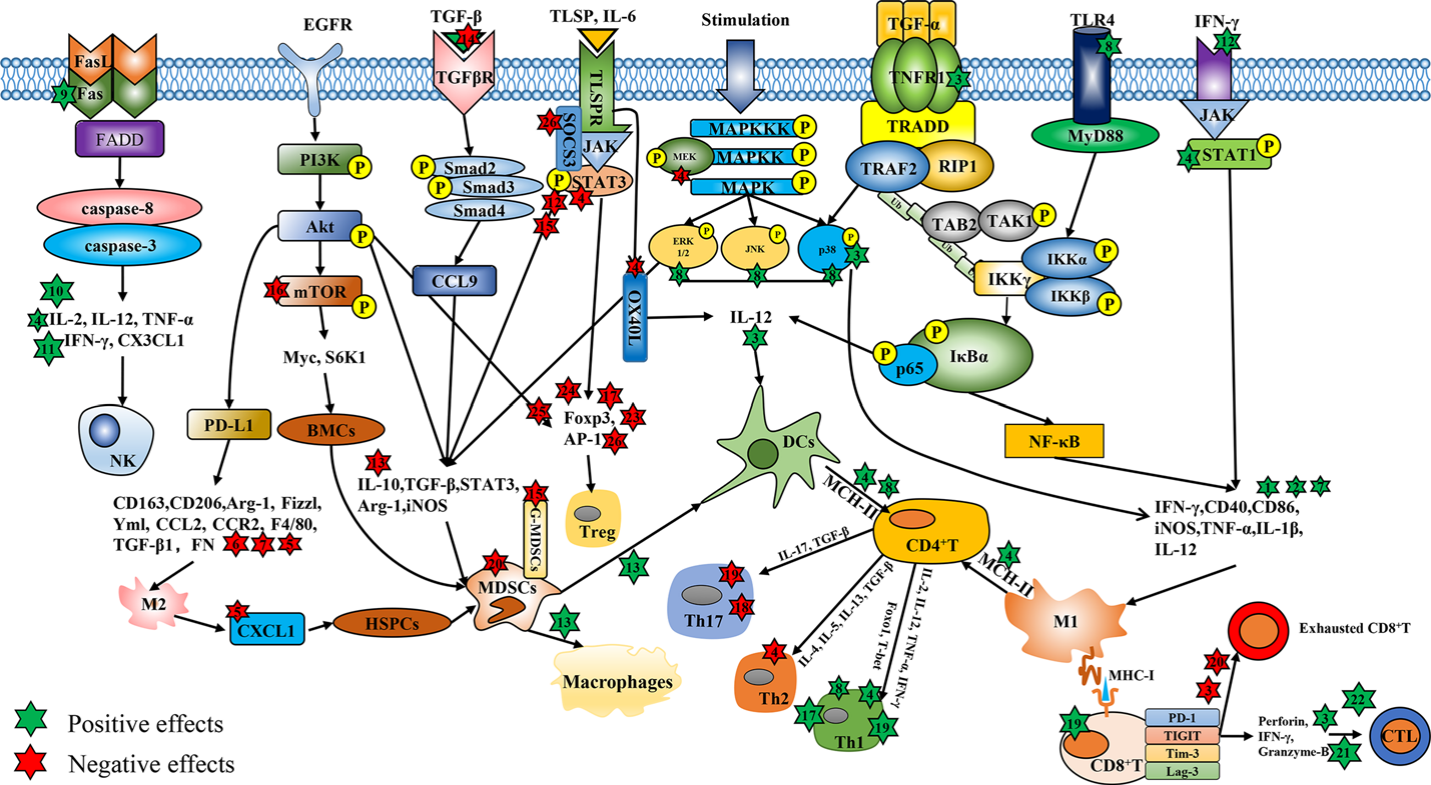
The regulatory effects of Chinese medicine compounds in immune cells. Chinese medicine compounds: 1. Modified Si-Jun-Zi Decoction; 2. Haimufang decoction; 3. Compound kushen injection; 4. Yu-ping-feng; 5. XIAOPI formula; 6. Dahuang Zhechong Pills; 7. Jianpi Yangzheng; 8. Yangyin Wenyang; 9. Jinfukang; 10. Tien-Hsien liquid; 11. ACNO; 12. Yanghe Decoction; 13. Jianpi Huayu Decoction; 14. Baoyuan Jiedu decoction; 15. Ze-Qi-Tang; 16. Shuangshen granules; 17. Quxie capsule; 18. Compound Sophorae Decoction; 19. JC-001 20. Shugan Jianpi formula; 21. Xiao-Ai-Ping; 22. Shenqi Fuzheng Injection; 23. Feiyanning Decoction; 24. Yi-Yi-Fu-Zi-Bai-Jiang-San; 25. Xihuang Pill; 26. Fuzheng Fangai.

**Figure 2:**
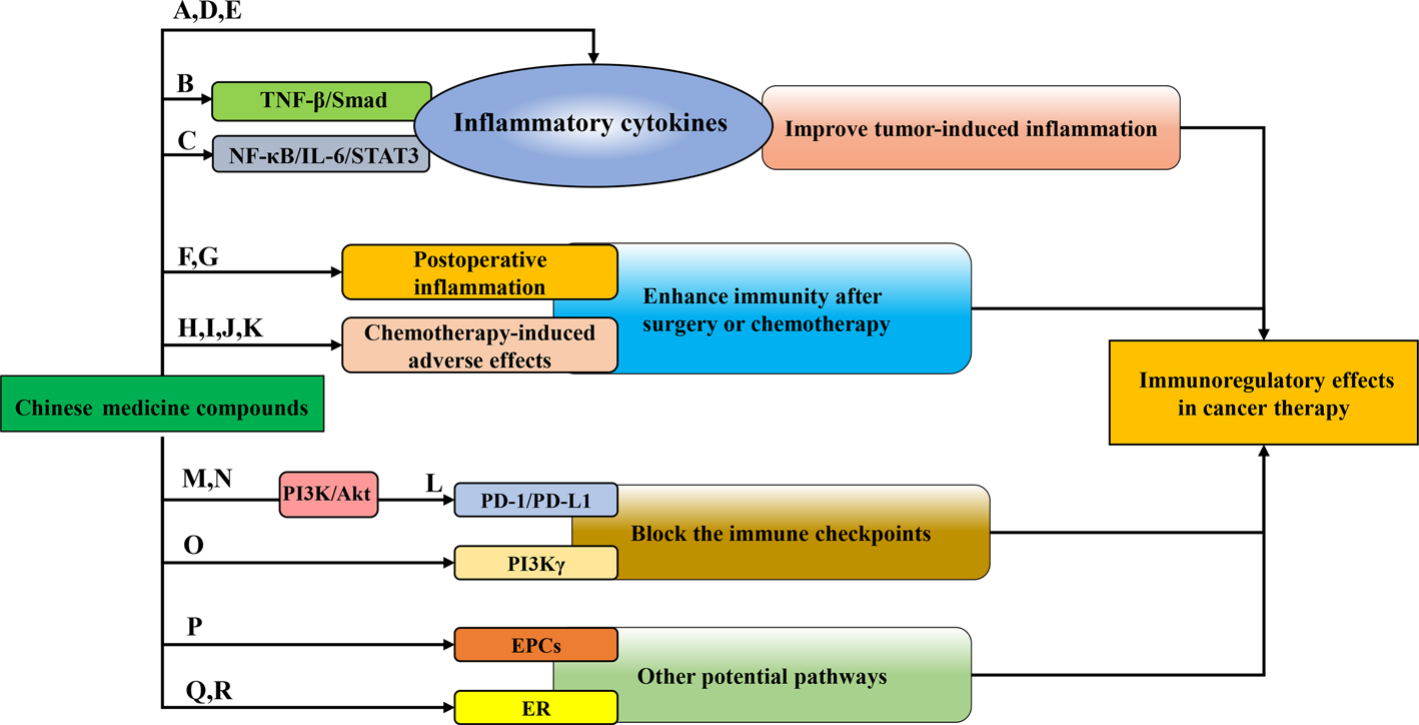
The immunoregulatory patterns of Chinese medicine compounds in cancer therapy. Chinese medicine compounds: **(A)** Banxia Xiexin decoction; **(B)** Compound kushen injection; **(C)** NHE-06; **(D)** Ruyiping+Platycodon grandiflorum; **(E)** Kejinyan decoction; **(F)** DahuangZhechong Pills; **(G)** San Huang decoction; **(H)** PHY906; **(I)** Ciji-Hua’ai-Baosheng; **(J)** Gegen Qinlian decoction; **(K)** Yangyin Fuzheng Decoction; **(L)** Bu Fei Decoction; **(M)** Huoxue Yiqi Recipe-2; **(N)** Bu-zhong-yi-qi decoction; **(O)** Modified Jian-pi-yang-zheng decoction; **(P)** Danggui Buxue decoction; **(Q)** XH formula; **(R)** Shugan Liangxue decoction.

Due to high morbidity and mortality, the malignant tumor has long been a hot issue in society. Surgery and chemotherapy are the primary treatments for cancers. Over the past decades, although researchers have devoted themselves to exploiting and exploring more effective treatments for cancers, the morbidity and mortality of malignant tumors are still high all over the world (1). Due to the heterogeneity and complexity of tumors, the cancer treatment focus from single target to multiple targets (2), and the tumor environment (TME) has attracted much attention. There, tumor-infiltrating immune cells play pivotal roles in tumor immunity and related functions. Chinese medicine compounds refer to the prescription composed of multiple kinds of traditional Chinese medicine (TCM) or the main components of TCM, which are used for treating malignant tumors given the characteristics of multi-pathways, multi-targets, slight side effects, and immunity enhancement (3). More and more Chinese medicine compounds have been recognized for their effects on tumor immunity, such as regulating the proliferation and activation of immune cells and their cytokines (4, 5). However, the impacts and mechanisms of Chinese medicine compounds on distinct immune cells and anti-cancer immunity are diverse and complicated. Herein, we provide an overview of the anti-cancer mechanisms of Chinese medicine compounds by regulating the diverse immune cells (**Figure 1**) and various underlying immunomodulatory pathways in cancer therapy (**Figure 2**).

## Immune Cells in Tumor Microenvironment

TME changes with tumor development and represents diverse immune cell composition and characteristics. Recently, immunotherapy becomes the most promising therapeutic strategy for cancer. In the TME, the functions of diverse immune cell populations function beneficially or harmfully through the local direct/indirect interaction with cancer cells. Typical immune cells in the TME include macrophages, dendritic cells (DCs), natural killer cells (NK cells), and myeloid-derived suppressor cells (MDSCs) in the innate immune system, as well as CD4^+^/CD8^+^T lymphocytes and regulatory T cells (Tregs) in the adaptive immune system. As the first defense line against pathogens, macrophages serve as typical antigen-presenting cells (APCs) with the functional capability of phagocytosis and antigen presentation. Tumor-associated macrophages (TAMs), which are recruited into tumors from monocytes in peripheral blood or macrophages in normal tissues, have been proven to assist tumor growth, angiogenesis, and metastasis, thus are considered as a promoter for tumor progression (6). In the TME, TAMs undergo differentiation with distinct properties in response to different stimuli, mainly M1 and M2 phenotypes. M2 macrophages that are activated by alternative pathways play a leading role in TAMs, which are capable of inducing cancer initiation and angiogenesis. Conversely, M1 macrophages are activated classically and have a strong ability of phagocytosis, antigen presentation, and secretion of various pro-inflammatory cytokines such as interleukin-6 (IL-6), IL-12, IL-23, tumor necrosis factor-α (TNF-α) and chemokines to effectively eliminate pathogens or tumor cells in the body (7). DCs are the strongest APCs in human immunity, which enable to efficiently ingest, process, and present antigens to naïve T lymphocytes to initiate adaptive immune responses, acting as a vital bridge linking innate immunity and adaptive immunity (8). Whereas, tumor-infiltrating DCs generally appear to be immature and functional paralysis, thereby failing to initiate or modulate adaptive immunity and gradually leading to immunosuppression (9). NK cells are the cytotoxic lymphocytes of the innate immune system. Although lack antigen receptors, they are regarded as the key cancer defensor due to the cytotoxic ability to quickly kill nearby target cells without antigen in advance. Similarly, there are a variety of endogenous mechanisms to resist NK cells from attack and deep infiltration, leading to the failed cytotoxicity (10). Moreover, MDSCs refer to a heterogeneous population of cells consisting of early myeloprogenitor cells, naïve granulocytes, as well as immature macrophages and DCs with various differentiation degrees, which display strong T-cell suppression function. Pro-inflammatory cytokines could induce the generation of MDSCs in tumor microenvironment and defect the differentiation of MDSCs into mature immune cells. Over the past years, numerous studies have shown that MDSCs could promote cancer progression in terms of cancer cell proliferation and invasion, tumor angiogenesis, and drug resistance (11).

As immunotherapy has been widely studied, CD4^+^ T lymphocytes, also known as helper T cells (Th), have attached much attention due to the remarkable effects on the adaptive immune system. The naïve T lymphocytes (Th0) differentiate into different subsets, such as Th1, Th2 and Th17. The Th1 immune response can be usually inhibited in a variety of malignant tumors, which manifests as the down-regulation of TNF-α, IL-2, IFN-γ and IL-2, and defecting the proliferation of cytotoxic T cells, macrophages and NK cells. On the contrary, Th2-biased immune response is up-regulated in cancer progression (12). The cytokines IL-4, IL-5 and IL-13 secreted by Th2 cells have been found to facilitate tumor growth (13). In addition, the ratio of Th1/Th2 refers to a crucial factor to maintain the normal immune state, and the imbalance of Th1/Th2 often occurs in immune-related diseases including cancer. Th17 is another helper T cell, which was named from the ability to secrete the pro-inflammatory cytokine IL-17. Th17 has been considered as a potential target for tumor treatment, however, in different tumors, Th17 showed inconsistent effects (14, 15). The subset of CD8^+^ T lymphocytes, also called cytotoxic T cells (CTLs), is an important guardian of the adaptive immune system. CTLs exert great cytotoxic effects on tumor cells, in other words, they perform cellular immunity by lysing or inducing apoptosis of targeted cells. However, with the infiltration of CD8^+^ T lymphocytes into tumor tissues, they undergo exhaustion in form of suppressed proliferation and even apoptosis (16). Tregs are a unique subset of CD4^+^CD25^+^ T lymphocytes and are augmented in various cancers. Tregs in the TME serve as a potent immunosuppressive factor and resist anti-tumor immunity (17). Additionally, the balance of Th17/Treg is likely to be disturbed by TAMs in the TME (18). Therefore, it is a feasible immunotherapeutic strategy to reshape TME by modulating the activity and function of immune cells.

## The Regulatory Effects of Chinese Medicine Compounds on Innate Immune Cells

### Macrophages

Targeted consumption or regulation of TAMs has become a potential cancer treatment strategy (19), such as activating TAMs towards M1-type polarization, suppressing M2-type polarization, and inducing M2 polarizing to M1. Numerous Chinese medicine compounds have been reported to exert inhibitory effects on tumor growth and metastasis through targeting macrophages (**Figure 1**). Th1 cytokines IFN-γ are conducive to M1 activation in the TME (20). Modified Si-Jun-Zi Decoction stimulated the secretion of GM-CSF, IFN-γ, IL-1α and IL-3, increased the number of macrophages, thus activating the innate immune system to remove colorectal cancer (CRC) cells (21). The elevated expression of phenotypic marker CD86 and CD40 stand for the activation of M1 macrophages to some extent. Ma et al. observed that Haimufang (HMF) decoction enhanced the generation of nitric oxide (NO) and reactive oxygen species (ROS), the secretion of cytokines TNF-α, IL-1β, IL-12 p70, and IL-6, and the expression of M1 marker CD40 and CD86, with time- and dose-dependent manners in RAW264.7 cells, but had no discernible impact on M2 phenotypic marker CD206. As a result, HMF promoted the M1 polarization and enhanced the phagocytosis ability (22). In hepatocellular carcinoma (HCC), Yang et al. reported that Compound kushen injection (CKI) facilitated the activation of macrophages by stimulating TNF receptor superfamily member 1 (TNFR1)-mediated NF-κB and p38 MAPK signaling pathways, thereby accelerating M1 polarization and alleviating TAMs-mediated immunosuppression (23). Yu-ping-feng (YPF) has usually been prescribed to enhance human immunity, which was reported to up-regulate the secretion of IL-1β and IL-12, the level of iNOS as well as the phosphorylation and activation of signal transducers and activators of transcription 1 (STAT1) in RAW264.7 cells to propel the M1 polarization and enhance M1-induced CD4^+^T cell activation and Lewis lung cancer (LLC) lysis (24). Furthermore, Chinese medicine compounds could restrain the function of M2 and facilitate the conversion of M2 to M1. XIAOPI (XP) formula remarkably prevented the promoting effect of TAMs on the proliferation and self-renewal activity of breast cancer cell lines (MDA-MB-231 and 4T1) in a co-culture system, and reversed the TAMs-mediated C-X-C Motif Chemokine Ligand 1 (CXCL1) secretion and β-catenin signal, thus attenuating the self-renewal activity and chemotherapy resistance of breast cancer stem cells (CSCs). Moreover, XP weakened the polarization of M2 by down-regulating the expression of CD163, CD206 and Arg1 as well as the transcriptional activity of CXCL1 in a dose-dependent manner (25). C-C Motif Chemokine Ligand-2 (CCL2) refers to an important chemokine for monocyte/macrophage chemotaxis (26). Chen et al. reported that CCL2 boosted the number of macrophages in the liver and induced M2 polarization, which could be prevented by Dahuang Zhechong Pills (DZP) through the suppressed secretion of CCL2 and its receptor CCR2, along with the low level of F4/80, TGF-β1 and FN to attenuate hepatic fibrotic status. DZP also reversed tumor-mediated M2 polarization of macrophages to reshape the immunosuppressive TME and inhibit the liver metastasis of CRC (27). Jianpi Yangzheng (JPYZ), the ingredients for invigorating qi and spleen of Jianpi Yangzheng Xiaozheng decoction, can promote the transformation of M2 to M1 with the elevation of CD86 and reduction of CD206 and CD163. The mRNA levels of M1-related genes (IL-1β, IL-12 and TNF-α) were enhanced by JPYZ while those of M2-related genes (Arg-1, Fizzl and Yml) were attenuated (28). Consequently, Chinese medicine compounds indeed regulate the activation and differentiation of TAMs to exert anti-tumor activity (**Table 1**).

**Table 1:** Innate immune cells impacted by Chinese medicine compounds.

Immune cells	Chinese medicine compounds	Signaling pathway/Cytokines	Specific main effects	Cancer types	Ref
Macrophages	Modified Si-Jun-Zi Decoction	↑GM-CSF, IFN-γ, IL-1α, IL-3,	↑Macrophages	Colorectal cancer	(21)
	Haimufang decoction	↑TNF-α, IL-1β, IL-12 p70, IL-6	↑Macrophages activity and M1	Lewis lung cancer	(22)
	Compound kushen injection	↑NF-κB and p38 MAPK signaling pathway	↑Macrophages activity and M1	Hepatocellular carcinoma	(23)
	Yu-Ping-Feng	↑STAT1 signaling pathway, IL-1β, IL-12, iNOS	↑The number and polarization of M1	Orthotopic lung cancer	(24)
	XIAOPI formula	↓TAMs/CXCL1 signaling pathway	↓Proliferation and polarization of M2	Breast cancer	(25)
	Dahuang Zhechong Pills	↓CCL2, CCR2, F4/80, TGF-β1, FN	↓Function and polarization of M2	Colorectal cancer	(27)
	Jianpi Yangzheng	↑IL-1β, IL-12, TNF-α; ↓Arg-1, Fizzl, Yml	↑The conversion of TAMs from M2 to M1	Gastric cancer	(28)
DCs	Yu-Ping-Feng	↑IL-12	↑The maturation and percentage of DCs	Hepatocellular carcinoma	(12)
	Yangyin Wenyang	↑MAPK, NF-κB signaling pathways	↑The number and maturation of DCs	Non-small cell lung cancer	(29)
	Compound kushen injection	↑IL-12	↑The killing activity of DC-cCSC FCs	Colorectal cancer	(30)
NK cells	Yu-Ping-Feng	↓TGF-β, Indoleamine 2,3-dioxygenase, IL-10; ↑IL-12	↑NK cell tumor infiltration and NK cell cytotoxicity	Lewis lung cancer	(31)
	Jinfukang	↑CX3CL1, TNF-α, Fas/FasL signaling pathway	↑The recruitment and cytotoxicity of NK cells	Lewis lung cancer	(32)
	Tien-Hsien liquid	↑IFN-γ, IL-2, TNF-α	↑The number and tumor-killing activities of NK cells	Colorectal cancer	(33)
	ACNO	↑IL-2, IL-12, INF-γ	↑The number and cytotoxicity of NK cells	Colorectal cancer	(34)
MDSCs	XIAOPI formula	↓TAMs/CXCL1 signaling pathway	↓The differentiation of HSPC into MDSCs	Breast cancer	(25)
	YHD	↓TGF-β and p-STAT3	↓The function of MDSCs		(35)
	Yu-Ping-Feng	↓Arg-1, iNOS, STAT3 ↓p-Akt, p-MEK, p-ERK, p-STAT3	↓The proportions of MDSCs subsets; ↑Apoptosis of MDSCs	Lewis lung cancer	(36)
	Jianpi Huayu Decoction	↓IL-10, TGF - β	↑The differentiation of MDSCs	Hepatocellular carcinoma	(37)
	Baoyuan Jiedu decoction	↓TGF-β/CCL9 signaling pathway	↓The content of G-MDSC and M-MDSC	Breast cancer	(38)
	Ze-Qi-Tang	↓STAT3/S100A9/Bcl-2/caspase-3 signaling pathway	↑The elimination of MDSCs (especially G-MDSCs)	Orthotopic lung cancer	(39)
	Shuangshen granules	↓mTOR/S6K1/Myc signaling pathway	↓The differentiation of BMCs into MDSCs	Lewis lung cancer	(40)

↑, Up-regulated or enhanced; ↓, Down-regulated or inhibited.

### Dendritic Cells (DCs)

Chinese medicine compounds could enhance the maturation and antigen presentation ability of DCs to induce the activation of naïve T lymphocytes and participate in the regulation of T cell differentiation (**Table 1**). IL-12 is closely involved in the antigen presentation function of DCs (41). Zhao et al. optimized a new traditional Chinese medicine formula Yangyin Wenyang (YYWY) on the basis of Jinfukang. YYWY intervention drove the impregnation of DCs, CD4^+^ and CD8^+^ T cells in LLC tissues, and raised the mRNA levels of IFN-γ, IL-1β, TNF-α and IL-12. In addition, YYWY stimulated MAPK and NF-κB signaling pathways in bone marrow dendritic cells (BMDCs) to accelerate the maturation of DCs and the proliferation and differentiation of T cells together (29). TSLP, an immune-related factor, can activate DCs and involve in T cell differentiation, which raises the expression of Th2-polarizing molecule CD252 (OX40L), and induces Th2 response (42). Yao et al. reported that YPF reduced the expression of TSLP and OX40L to induce the maturation of DCs and the expression of CD80, CD86, MHC-II as well as IL-12 (12). Furthermore, DCs combine with tumor cells to form fusion cells (FCs), which play an inhibitory role in tumor development. Yang et al. found that fusion cells of dendritic colon cancer stem cells (DC-cCSC FCs) facilitated the proliferation of cytokines induce killer (CIK) cells. The cytotoxicity of DC-cCSC FCs and CIK cells against cCSCs was further improved by IL-12 and could be enhanced by the Chinese medicine CKI (30). In summary, Chinese medicine compounds are capable to activate DCs. Although some studies have proven that Chinese medicine compounds elevate the function of NK cells in the TME by promoting the secretion of IL-12 (**Figure 1**), more researches about the mechanisms are demanded.

### Natural Killer Cells (NK Cells)

Recently, the anti-tumor activity of exogenously activated and amplified NK cells has been proved in clinical (43). Some studies have shown that activating NK cells may be an effective approach for Chinese medicine compounds to suppress the advance of tumors (**Table 1**). For example, Luo et al. confirmed that YPF propelled the infiltration, proliferation, and killing activity of NK cells in tumor tissues to prolong the survival of LLC tumor-bearing mice. Elimination of NK cells could remarkably reverse the inhibitory effect of YPF on lung cancer, indicating that NK cells were the potential target cells for YPF-mediated lung cancer treatment (31). Additionally, Que et al. found that Jinfukang was conducive to the secretion of C-X3-C Motif Chemokine Ligand 1 (CX3CL1) in circulating tumor cells (CTCs) and the recruitment of CTCs to NK cells. Meanwhile, Jinfukang significantly accelerated NK cells-mediated CTCs apoptosis and prevented lung cancer metastasis through activating Fas/FasL signaling pathway, manifested as the up-regulated FasL and secretion of TNF-α in NK cells (32). In addition, Tien-Hsien liquid (THL) accelerated the secretion of immune factors including IFN-γ, IL-2 and TNF-α to augment the cytotoxicity of NK cells and CTLs and mitigate the tumor development (33). Finally, Li et al. reported a new traditional Chinese medicine compound anti-cancer No.1 (ACNO) which could dose-dependently enhance the cytotoxicity of NK cells by up-regulating the secretion of IL-2, IL-12, and INF-γ (34) (**Figure 1**). In brief, Chinese medicine compounds can interfere with tumor growth by the way of enhancing the killing activity of NK cells.

### Myeloid-Derived Suppressor Cells (MDSCs)

Researches have suggested that Chinese medicine compounds abated the immunosuppression in the TME by facilitating the apoptosis and differentiation of MDSCs to enhance immune response (**Table 1**). Mao et al. observed that Yanghe Decoction (YHD) had immunomodulatory effects on 4T1 breast tumors, not only boosting the amount of NKT and CD4^+^ T cells as well as the secretion of IFN-γ and p-STAT1, but also attenuating the recruitment of MDSCs through JAK/STAT3 signaling pathway (35). YPF suppressed the mRNA levels of immunosuppressive genes in MDSCs, including Arg-1, iNOS and STAT3, and decreased the levels of p-Akt, p-MEK, p-ERK and p-STAT3 to prevent MDSCs augment and induce their apoptosis, which further modulated the proportion of T cell subsets by increasing CD4^+^/CD8^+^ T lymphocytes and decreasing Treg proportion, thus reshaping the immune TME and preventing the lung cancer progression (36). Moreover, Xie et al. proved that Jianpi Huayu Decoction (JHD) attenuated the expression of IL-10 and TGF-β in tumor tissues and accelerated the differentiation of MDSCs into macrophages and DCs. JHD decreased the content of ROS in MDSCs, inhibited CD4^+^ T cells proliferation and the percentages of Th17 and Treg, but elevated the proportion of CTLs and DCs to relieve the MDSCs-mediated immunosuppressive state in HCC (37). Another research reported that Baoyuan Jiedu decoction (BYJD), a traditional Chinese medicine formula composed of Astragalus, Ginseng, Aconite root, Honeysuckle, Angelica, and Licorice, inhibited the TGF-β/CCL9 signaling pathway and reduced the number of MDSCs in peripheral blood and spleen of breast cancer (4T1)-bearing mice. Furthermore, BYJD blocked the recruitment of MDSCs in lung, the metastatic organ of breast cancer, thereby improving the pre-metastatic niche (PMN) and prolonging the survival of tumor-bearing mice (38). Xu et al. found that the medium dose of Ze-Qi-Tang (ZQT) had the strongest anti-tumor activity. In lung carcinoma *in situ*, ZQT could eliminate MDSCs in a dose-dependent manner, reduce the recruitment of MDSCs, and enhance the infiltration of T cells to reverse MDSCs-mediated immunosuppression. Thoroughly discovered that ZQT suppressed the level of STAT3, p-STAT3 and anti-apoptotic protein Bcl-2, increased the expression of pro-apoptotic protein Bax, cleaved caspase-3 and PARP to induce the apoptosis of G-MDSCs in lung carcinoma *in situ*. However, after G-MDSCs were completely depleted, the cytotoxicity of CD8^+^ T cells and the inhibitory effect of MDSCs had no significant difference between ZQT group and the control group, suggesting that the anti-tumor activity of ZQT was achieved by targeting G-MDSCs subset (39). Chinese medicine compounds also could decline the generation of MDSCs. Shuangshen granules restrained the expression of mTOR, S6K1 and Myc to block the differentiation of bone marrow cells (BMCs) into MDSCs in a dose-dependent manner, thus preventing lung metastasis (40). XP impaired the activation of hematopoietic stem-progenitor cells (HSPCs) as well as the differentiation of HSPCs into MDSCs through TAMs/CXCL1 signaling pathway to inhibit lung metastasis of breast cancer (25). Therefore, it may be a promising therapeutic way for Chinese medicine compounds to improve TME through targeting MDSCs (**Figure 1**).

## The Regulatory Effects of Chinese Medicine Compounds on Adaptive Immune Cells

### CD4^+^ T Lymphocytes

Some Chinese medicine compounds are capable of exerting anti-tumor effects through facilitating CD4^+^T lymphocytes differentiate to Th1 and attenuating Th2 response (**Table 2**). The activation and differentiation of CD4^+^ T lymphocytes tend to be affected by APCs, such as macrophages and DCs. YPF enhanced the antigen presentation ability of macrophages by up-regulating the level of MHC II, thereby activating the LLC cell lysis mediated by CD4^+^ T cells. Meanwhile, YPF enhanced the secretion of Th1 cytokines (IL-2, IL-12) but repressed the level of Th2 cytokines (TGF-β, IL-4) (24). In addition, in H-22 tumor-bearing mice, YPF elevated the ratio of Th1/Th2 (IFN-γ/IL-4) through mature DCs, thus ameliorating Th2-biased immune state (12). Similarly, YYWY exerted the anti-tumor effect by inducing DCs mature, which subsequently activated CD4^+^ T lymphocytes and enhanced Th1 function (29). Chen et al. found that in CT26 tumor-bearing mice, Quxie capsule (QX) intervention boosted the expression of Foxo1 and T-bet, along with the augment of Th1 and Th1/Th2, to propel the immune response in TME (44). Th17 has been considered as a potential target for tumor treatment in several studies. Deng et al. confirmed that Th17 was conducive to the occurrence of Ulcerative Colitis-Related Colorectal Cancer (UCRCC), however, Compound Sophorae Decoction (CSD) remarkably reversed the increasing number of Th17 and the secretion of IL-17 to relieve inflammation in UCRCC (45). Chuang et al. reported that in the co-culture system of spleen cells and LLC cells, JC-001 enhanced Th1 function and suppressed Th17 function by abating the secretion of TGF-β and IL-17A to prevent tumor progression (46). In liver cancer, JC-001 had the same effects on Th1 and Th17 function, thereby alleviating Treg-mediated immunosuppression (47). Taken together, Chinese medicine compounds control tumor growth by modulating the differentiation of CD4^+^ T cells, enhancing Th1 immune response as well as restraining the function of Th2 and Th17 (**Figure 1**).

**Table 2:** Adaptive immune cells impacted by Chinese medicine compounds.

Immune cells	Chinese medicine compounds	Signaling pathway/Cytokines	Specific main effects	Cancer types	Ref
CD4^+^ T lymphocytes	Yu-Ping-Feng	↑IFN-γ/IL-4 ratio↑IL-12, TNF-α, IFN-γ; ↓IL-10, IL-5, IL-4	↓Th2-biased immune state	Hepatocellular carcinoma	(12)
		↑MHC II in Macrophages; ↑IL-2, IL-12; ↓TGF-β, IL-4	↑Th1 immune response; ↑The lysis capacity of CD4^+^T cells	Orthotopic lung cancer	(24)
			↑The proportion of CD4^+^T cells	Lewis lung cancer	(36)
	Yangyin Wenyang	↑TNF-α,IL-2,IFN-γ; ↑IFN-γ/IL-4 radio	↑The differentiation of T cells into Th1; ↑The ratio of Th1/Th2	Non-small cell lung cancer	(29)
	Quxie capsule	↑Foxo1, T-bet	↑The ratio of Th1/Th2	Colorectal cancer	(44)
	Compound Sophorae Decoction	↓STAT3, IL-17	↓The proportion of Th17	UCRCC	(45)
	JC-001	↑IL-2, IL-10, TNF-α, IFN-γ; ↓TGF-β, IL-17A	↑Th1 function; ↓Th17 function	Lewis lung cancer	(46)
		↑T-bet, GATA-3	↑Th1 function; ↓Th17 function	Hepatocellular carcinoma	(47)
CD8^+^ T lymphocytes	Compound kushen injection	↓Lag-3, PD-1, TIGIT,Tim-3;	↓CD8^+^T cells exhaustion;	Hepatocellular carcinoma	(23)
		↑TNF-α, Perforin, IFN-γ, Granzyme-B	↑CD8^+^T cells cytotoxicity		
	Yangyin Wenyang	↑MAPK, NF-κB signaling pathways	↑CD8^+^T cells generation by mature DCs	Non-small cell lung cancer	(29)
	Tien-Hsien liquid	↑IFN-γ, IL-2, TNF-α	↑The number and cytotoxicity of CTLs	Colorectal Cancer	(33)
	Yu-Ping-Feng		↑The proportion of CD8^+^T cells	Lewis lung cancer	(36)
	JC-001	↑IL-12 p70, IFN-γ	↑CD8^+^T cells generation	Lewis lung cancer	(46)
	Shugan Jianpi formula		↓CD8^+^T cells apoptosis	Breast cancer	(48)
	Xiao-Ai-Ping	↑Perforin, IFN-γ, Granzyme-B	↑CD8^+^T cells proliferation and function	Lewis lung cancer	(49)
	Shenqi Fuzheng Injection	↓IL-10, TGF-β, VEGF	↑The cytotoxic and migratory activities of Jurkat T cells	Melanoma	(50)
Tregs	Quxie capsule	↓Foxp3 induced by↑Foxo1	↓The proportion of Treg; ↓The ratio of Th17/Treg cells	Colorectal cancer	(44)
	JC-001	↓Foxp3, RORγt	↓Treg function	Hepatocellular carcinoma	(47)
	Feiyanning Decoction	↓Foxp3	↓The number of Tregs	Lewis lung cancer	(51)
	Yi-Yi-Fu-Zi-Bai-Jiang-San	↑IL-6, CCXL13, IL-10	↓Tregs activated by ETBF	Colorectal cancer	(52)
	Xihuang Pill	↓PI3K/Akt/AP-1 signaling pathway	↓The proliferation of Tregs; ↑The apoptosis of Tregs	Breast cancer	(53)
	Fuzheng Fangai	↓IL-17, IL-23, IFN-γ, Foxp3, RORγt, SOCS3, JAK2, STAT3	↓The proportion of Th17 and Treg; Restore the balance of Th17/Treg	Lewis lung cancer	(54)

↑, Up-regulated or enhanced; ↓, Down-regulated or inhibited.

### CD8^+^ T Lymphocytes

Restoring CD8^+^ T lymphocytes function has always been regarded as an effective method to facilitate tumor immunity (16). Some studies have demonstrated that Chinese medicine compounds lead to the augment of CD8^+^ T lymphocytes in tumor tissues (29, 33, 36). In addition, Chinese medicine compounds bring vital impacts on the activity and exhaustion of CD8^+^ T cells (**Table 2**). CKI combined with sorafenib dramatically suppressed function-inhibitory receptors in CD8^+^ T cells, including lymphocyte-activation gene 3 (Lag-3), programmed cell death protein 1 (PD-1), T-cell immunoreceptor with Ig and ITIM domains (TIGIT), and T-cell immunoglobulin and mucin-domain containing-3 (Tim-3) to reduce the depletion of CD8^+^ T cell. At the same time, the combination therapy strengthened the cytotoxicity of CD8^+^ T cells by accelerating the production of TNF-α, Perforin, IFN-γ and Granzyme-B. When CD8^+^ T lymphocytes were blocked, the anti-tumor activity of combination therapy disappeared, suggesting that CD8^+^ T cells were necessary for CKI-mediated HCC treatment (23). Lu et al. reported that, in depression breast cancer mice, Shugan Jianpi formula combined with chemotherapy drug gemcitabine (GEM) effectively repressed the apoptosis of CD8^+^ T cells and significantly reduced MDSCs to enhance the immune surveillance, thereby preventing the progress of breast cancer (48). It has been shown that Xiao-Ai-Ping, an adjuvant injection for tumor therapy, propelled the infiltration and function of CD8^+^ T cells in the TME of LLC, mainly manifested as the increase in Perforin, IFN-γ and Granzyme-B (49). At last, JC-001 drove the generation of CD8^+^ T cells in LLC1 tumor and the secretion of IL-12 p70 and IFN-γ to sensitize LLC1 tumor to chemotherapeutic drug cisplatin (CDDP) (46). Jurkat T cell line, a kind of CTLs, has been reported to resist tumor immune escape (55). Du et al. observed that Shenqi Fuzheng Injection (SFI) down-regulated the immunosuppressive cytokines IL-10, TGF-β and VEGF in A375 cells in a concentration-dependent manner. Also, SFI enhanced the cytotoxic and migratory activities of Jurkat T cells in A375 melanoma to reprogram the melanoma microenvironment (50), which demonstrated that CTLs can mediate the anti-tumor effects of Chinese medicine compounds. In conclusion, the restoration and enhancement of the activity of CD8^+^ T cells are effective ways for Chinese medicine compounds to prevent cancer from deteriorating (**Figure 1**).

### Regulatory T Cells (Treg)

Previous researches have shown the potential therapeutic effects of targeting Foxp3^+^ Tregs for cancer treatment (56). Guo et al. reported that Feiyanning Decoction significantly attenuated the amount of CD4^+^CD25^+^ regulatory T cells in spleen, thymus and tumor tissues in LLC-bearing mice. It decreased the mRNA level of Foxp3, enhanced tumor immune response, and slowed down the progression of lung cancer (51). Yi-Yi-Fu-Zi-Bai-Jiang-San (YYFZBJS), a classical Chinese medicine formula for gastrointestinal diseases, suppressed the number of CD4^+^CD25^+^ Foxp3^+^ T cells. Although YYFZBJS did not directly affect CRC cells, it was able to down-regulate the proliferation of MC-38 cells through Enterotoxigenic Bacteroides fragilis (ETBF)-primed Tregs and preventing the advance of colon cancer (52). Moreover, Xihuang (XH) Pill reduced the proliferation of Tregs in the TME of breast cancer and induced the apoptosis of Tregs by suppressing PI3K/Akt/AP-1 signaling pathway, thus reversing the immune escape to attenuate the tumor growth (53). The imbalance of Th17/Treg often occurs in autoimmune diseases and inflammation (57), which could be effectively ameliorated by Chinese medicine compounds. CSD restored the balance of Th17/Treg by decreasing the proportion of Th17, Treg and the related inflammatory factors to improve immune function (58). Inflammation is often closely associated with the advance of cancer, thereby adjusting Th17/Treg balance in cancer may be helpful to relieve tumor-mediated immunosuppression. On the one hand, QX down-regulated Foxo1-mediated Foxp3, reduced Tregs and recovered the balance of Th17/Treg, thus playing a therapeutic role in CRC (44). On the other hand, Liu et al. found that Fuzheng Fangai combined with cyclophosphamide (CTX) effectively decreased the proportion of Th17 and Treg in spleen and tumor tissues of LLC tumor-bearing mice and restored the ratio of Th17/Treg. Meanwhile, the secretion of IL-17, IL-23 and IFN-γ, the mRNA levels of Foxp3 and RORγt as well as the protein levels of SOCS3, JAK2, and STAT3 were remarkably down-regulated by combined therapy compared to CTX alone (54). Summarily, Chinese medicine compounds are capable of suppressing tumor development through the inhibitory effects on Tregs (**Table 2**) (**Figure 1**).

## The Immunoregulatory Patterns of Chinese Medicine Compounds in Cancer Therapy

### Improve Tumor-Induced Inflammation

Chronic inflammation serves as a risk factor for cancers. Tumor-induced inflammation accelerates the enrichment and activity of inflammatory cells in tumor tissue, thereby promoting tumor progression (59). Therefore, it may be feasible to treat cancers through improving inflammation to enhance immune function by Chinese medicine compounds (**Figure 2**). CRC was the third common cancer around the world, the morbidity of which ranked the second. Statistically, the incidence of CRC was higher in transition countries (1). Banxia Xiexin decoction (BXD) has been often applied to treat a variety of inflammatory diseases, including acute and chronic gastritis, oral ulcer, digestive ulcer, and so on. Yan et al. found that BXD enhanced the serum level of pro-inflammatory cytokines IL-1β, TNF-α and IL-6 in colon cancer-bearing nude mice and improve inflammation (60). With the popularization of the hepatitis B vaccine, the total incidence and mortality of liver cancer have been declining in the past three decades, whereas primary liver cancer still continues. HCC is the most common primary liver cancer (1). Hepatic fibrosis is the most vital hazard factor of HCC (61). Yang et al. established chronic liver fibrosis models with CCl_4_ or MCD and found that CKI remarkably attenuated the infiltration of macrophages in the livers of these mice, down-regulated the expression of TNF-α and IL-6, thus alleviating the inflammatory response induced by liver fibrosis. Furthermore, Smad7 was identified as a key target of CKI in the treatment of liver fibrosis. CKI up-regulated Smad7, inhibited TNF-βR1, and suppressed TNF-β/Smad signaling pathway to prevent the development of chronic liver fibrosis and HCC (62). NHE-06 exerted a strong anti-inflammatory activity on HCC through the suppression of NF-κB/IL-6/STAT3 signaling and TGF-α and PTSG2. Meanwhile, NHE-06 strengthened the anti-tumor immunity by increasing the infiltration of immune cells and the production of IFN-γ. However, the preventive and therapeutic efficacy against HCC could be merely realized in mice with an intact immune system (63). Breast cancer has become the most common malignant tumor in the world and one of the main causes of death in females (1). Ruyiping (RYP) has been reported as a traditional Chinese medicine formula for breast cancer metastasis. Ye et al. found that in breast cancer 4T1-bearing mice, RYP combined with Platycodon grandiflorum (RP) effectively decreased the content of inflammatory marker Fibrinogen and inflammation-associated cytokines IL-6 and IL-1β in the lung tissue, improved the pulmonary inflammatory microenvironment resulted from breast cancer, and prevented the lung metastasis of breast cancer (64). According to Global Cancer Statistics 2020, lung cancer has become the most deadly malignant tumor in the world, whose morbidity ranked second among all cancers (1). Kejinyan decoction is an empirical prescription of traditional Chinese medicine for lung cancer clinically. Chen et al. reported that, after intervention with Kejinyan decoction, the inflammatory cytokines TNF-α, IFN-γ, IL-6, IL-4 and IL-13 were reduced and the survival of LLC tumor-bearing mice was prolonged (65). In short, Chinese medicine compounds could regulate the secretion of inflammatory cytokines to improve the inflammatory response in cancers.

### Enhance Immunity After Surgery or Chemotherapy

Surgery and chemotherapy are the common treatments for cancers. However, side effects are always the major concern and limitation. Surgery tends to trigger inflammation, which leads to various unfavorable prognoses. Chemotherapy can damage the immune system even more. Therefore, Chinese medicine compounds combined with surgery or chemotherapy are typically used in clinical treatments to enhance efficacy and reduce side effects (**Figure 2**). Transcatheter arterial chemoembolization (TACE) has been identified as one of the therapies for HCC (66). Dai et al. reported DZP combined with TACE enhanced the immune functional indexes in serum and the number of CD4^+^/CD8^+^ T cells and reduced VEGF, TGF-β1 and MMP-2 to ameliorate cancer metastasis and other adverse reactions after TACE (67). A clinical trial has demonstrated that San Huang decoction remarkably reduced the volume of exudate after breast cancer surgery and the expression of TNF-α, IL-6, IL-8 and C-reactive protein (CRP) to improve the inflammation. Some studies have illustrated that Chinese medicine compounds could not only enhance the effectiveness of chemotherapy (23, 46, 48), but also alleviate the side effects of chemotherapy, including diarrhea, weight loss and other symptoms of hypoimmunity. PHY906 has been utilized for thousands of years to treat gastrointestinal diseases. In recent years, it has been developed as an adjuvant for cancer. Lam et al. found that PHY906 was capable of accelerating the infiltration of macrophages into tumors by inducing the up-regulation of macrophage cytokines hMCP1, dramatically promoted the conversion of macrophages to M1, and enhanced the anti-tumor effects of Sorafenib (68). In addition, Ciji-Hua’ai-Baosheng improved the immune function of H-22 tumor-bearing mice after treatment with chemotherapy and alleviated CTX-induced colitis, manifested as elevated lymphocytes in spleen, augmented IL-2, IFN-γ and TNF-α, and the reduced IL-6 in serum and tumor tissues (69). Similarly, Wu et al. found Gegen Qinlian decoction (GQT), a classic Chinese medicine compound widely applys to gastrointestinal inflammatory diseases, assisted the anti-tumor effects of irinotecan (CPT-11) on colon cancer. It reversed the abnormal enhancement of IL-1β, COX-2, ICAM-1, and TNF-α. Moreover, GQT relieved diarrhea in CPT-11-treated patients by restraining hCE2 (70). Tumor suppressor p53 has been considered to be able to regulate immunity. Inactivated p53 may influence the effects of BMCs and T cells (71). Wei et al. reported that Yangyin Fuzheng Decoction promoted the infiltration of inflammatory cells to improve anti-tumor immunity and restore the CDDP-mediated weight loss of mice (72). Thus, Chinese medicine compounds combined with conventional therapy are expected to be a promising treatment for patients suffering from malignant tumors.

### Block the Immune Checkpoints

Immune checkpoints represent a series of molecules on immune cells, which are recognized as switches of immune functions. In the TME, immune checkpoints are often overexpressed or activated, leading to the paralysis of the immune system. PD-1 and its ligand 1 (PD-L1) are the pivotal immune checkpoints that promote immune escape and cancer advance. Targeting the PD-1/PD-L1 axis has been effective immunotherapy against cancer (73). The combined therapy of GQT and PD-1 blocker effectively accelerated the proliferation of CD8^+^ T cells and restored the T-cell function by up-regulating IL-2 and IFN-γ. Moreover, some Chinese medicine compounds themselves exerted blocking effects on immune checkpoints. Bu Fei Decoction adjusted the immunosuppressive TAMs in non-small cell lung cancer (NSCLC) and inhibited its tumor-promoting effect by down-regulating IL-10 and PD-L1 (74). Studies have found that the PI3K/Akt signaling pathway assisted the activation of PD-1/PD-L1 axis. Teng et al. summarized that the active ingredients of Huoxue Yiqi Recipe-2 (HYR-2) targeted PD-L1 signaling pathway in the treatment of lung cancer. HYR-2 could turn M2 to M1 through down-regulating PD-L1 that is closely associated with the blockage of PI3K/Akt signaling pathway (75). Xu et al. observed that Modified Bu-zhong-yi-qi decoction suppressed the expression of PD-L1 through blocking PI3K/Akt signaling pathway in gastric cancer, thereby up-regulating the ratio of CD4^+^/CD8^+^ T cells and the number of CD8^+^PD-1^+^ T cells as well as decreasing the proportion of PD-1^+^ Tregs induced by chemotherapy, representing as a promising therapy for gastric cancer (76). PI3Kγ is an immune checkpoint for macrophages, which can switch the polarization of macrophages to reshape immune microenvironment and control immune suppression in cancers (77). Yuan et al. reported that Modified Jian-pi-yang-zheng decoction down-regulated PI3Kγ remarkably, promoted the secretion of TNF-α and IL-1β while decreased IL-10, accelerated the conversion of M2 to M1 and the differentiation of TAMs, thus prevented the progression and metastasis of gastric cancer (78) (**Figure 2**). Although immune checkpoint has gained great attention in tumor immunotherapy, the impacts of Chinese medicine compounds on immune checkpoints are not fully investigated.

### Other Potential Ways

Recently, erythroid progenitor cell (EPC) has been known as the regulator in tumor immune response. In malignant tumors, EPCs did not differentiate into mature red blood cells and have inhibitory effects on tumor immunity (79). Li et al. reported that Danggui Buxue decoction reduced the abnormal accumulation of EPCs in melanoma and accelerated EPCs to differentiate into mature red blood cells, which led to relieving anemia, enhancing tumor immune response, and inhibiting the progression of melanoma (80). Estrogen/estrogen receptor (ER) has also been considered as a possible immunotherapy target for cancers (81). There are two forms of ER, ERα and ERβ. When the increase in ERα level leads to the imbalanced ratio of ERα/ERβ or the mutation rate of ERα augments, it is more likely to induce breast cancer. XH formula has been used for the treatment of breast cancer since 1740, which suppressed the proliferation and activity of breast cancer cells in dose- and time-dependent manners. Hao et al. reported that XH formula bond to ERα and HSP90, promoting the disintegration of ERα, and blocking the transmission of ERα signaling. Through this anti-estrogen-like effect, XH formula inhibited the progression of breast tumors (82). Moreover, it was reported that Shugan Liangxue (SGLX) decoction suppressed the protein levels of ERα and 17β-estradiol (E2) target genes, c-Myc and Bcl-2, in human breast cancer cells. It was speculated that SGLX exerted inhibitory effects on ER^+^ breast cancer cells selectively (83) (**Figure 2**).

## Perspectives

There are various ways of Chinese medicine compounds to exert anti-tumor activity, including blocking cell cycle, inhibiting cell viability and proliferation, inducing cell apoptosis, preventing cell invasion and migration, and enhancing the sensitivity of tumor cells to chemotherapy drugs, etc. In recent years, immunotherapy has become the most promising field in cancer therapy. Regulating immune cells has been proved to be a powerful weapon against cancers and is increasingly applied clinically. Traditional Chinese medicine or its extracts have been reported to play a variety of roles in immune cells, thereby enhancing innate and adaptive immunity (84, 85). By the advantages of multi-components and multi-targets, Chinese medicine compounds have more comprehensive effects and mechanisms of regulating tumor immunity. Traditional Chinese medicine compound preparations, such as traditional Chinese medicine patent prescription and Chinese medicine compound injection, have been put into application clinically due to their anti-tumor and anti-inflammatory activity. The studies on mechanisms of Chinese medicine compounds enhancing tumor immunity mostly concentrated on the number and activity of immune cells in the TME, however, more in-depth and systematic researches are still needed and worth being further explored, including but not limited to: the targeted immune cell types which were regulated specifically by Chinese medicine compounds, the direct targets and pathways, either in immune cells or cancer cells, which mediated the immunotherapeutic effects of Chinese medicine compounds, the different immunomodulatory effects in distinct TME, the combined effect of immunoregulating Chinese medicine compounds with chemotherapy or immunotherapy drugs. Furthermore, the roles of various components in Chinese medicine compounds and their interplay or relationship in different symptoms is another important question. Nowadays, researchers try to depict formulas of traditional Chinese medicine compounds and optimize new formulations with more active ingredients and better therapeutic effects. Therefore, it is necessary to carry out more systematic researches on the immunoregulatory and pharmacological effect of TCM on cancer therapy and the underlying mechanisms to provide the more comprehensive theoretical basis for the clinical application of Chinese medicine compounds in cancer treatment.

## Author Contributions

FC drafted the manuscript. JL, HW and QB drafted and revised the manuscript. All authors contributed to the article and approved the submitted version.

## Funding

This study was supported by grants from the National Natural Science Foundation of China (81630086, 82030099, and 81973078), National Key R&D Program of China (2018YFC2000700), Shanghai Municipal Human Resources and Social Security Bureau (2018060), Shanghai Public Health System Construction Three-Year Action Plan (GWV-10.1-XK15), and Innovative Research Team of High-Level Local Universities in Shanghai.

## Conflict of Interest

The authors declare that the research was conducted in the absence of any commercial or financial relationships that could be construed as a potential conflict of interest.

## Publisher’s Note

All claims expressed in this article are solely those of the authors and do not necessarily represent those of their affiliated organizations, or those of the publisher, the editors and the reviewers. Any product that may be evaluated in this article, or claim that may be made by its manufacturer, is not guaranteed or endorsed by the publisher.

## Glossary

ACNO: Anti-cancer No.1
Akt: Protein kinase B
AP-1: Activator protein-1
APCs: Antigen presenting cells
Arg-1: Arginase-1
Bax: Bcl2-Associated X
Bcl-2: B-cell lymphoma-2
BMCs: Bone marrow cells
BMDCs: Bone marrow dendritic cells
BXD: Banxia Xiexin decoction
BYJD: Baoyuan Jiedu decoction
CCL2/CCR2: Chemokine (C-C motif) ligand 2/C-C chemokine receptor type 2
CCl_4_: Carbon tetrachloride
CCL9: Chemokine (C-C motif) ligand 9
CDDP: Cisplatin
CIK cells: Cytokines induce killer cells
CKI: Compound kushen injection
COX-2: Cyclooxygenase-2
CPT-11: irinotecan
CRC: Colorectal cancer
CRP: C-reactive protein
CSCs: Cancer stem cells
CSD: Compound Sophorae Decoction
CTCs: Circulating tumor cells
CTL/Tc: Cytotoxic T cell
CTX: Cyclophosphamide
CXCL1: Chemokine (C-X-C motif) Ligand 1
CX3CL1: Chemokine (C-X3-C motif) Ligand 1
DCs: Dendritic cells
DC-cCSC FCs: Fusion cells of dendritic colon cancer stem cells
DZP: Dahuang Zhechong Pills
E2: Estradiol
EPC: Erythroid progenitor cell
ER: Estrogen receptor
ERK: Extracellular regulated protein kinases
ETBF: enterotoxigenic bacteroides fragilis
Fas/FasL: Factor associated suicide/Fas Ligand
FCs: Fusion cells
FN: Fibronectin
Foxo1: Forkhead box O1
Foxp3: Forkhead box p3
GEM: gemcitabine
GM-CSF: Granulocyte-macrophage colony stimulating factor
GQT: Gegen Qinlian decoction
HCC: Hepatocellular carcinoma
hCE2: human carboxylesterase 2
hMCP1: Human monocyte chemoattractant protein-1
HMF: Haimufang
HSPCs: Hematopoietic stem-progenitor cells
HSP90: Heat shock protein 90
HYR-2: Huoxue Yiqi Recipe-2
ICAM-1: Intercellular adhesion molecule-1
IFN-γ/INF-γ: Interferon-gamma
IL: Interleukin
iNOS: Inducible nitric oxide synthase
JAK: Janus Kinase
JHD: Jianpi Huayu Decoction
JPYZ: Jianpi Yangzheng
Lag-3: Lymphocyte-activation gene 3
LLC: Lewis lung cancer
MAPK: Mitogen-activated protein kinase
MCD: Methionine-choline deficient
MDSCs: Myeloid-derived suppressor cells
MEK: Mitogen-activated protein kinase kinase
MHC: Major histocompatibility complex
MMP: Matrix metalloproteinase
mTOR: Mechanistic target of rapamycin
Myc: Myelocytomatosis oncogene
NF-κB: nuclear factor kappa-B
NK cells: Natural killer cells
NKT cells: Nature killer T cells
NO: nitric oxide
NSCLC: Non-small cell lung cancer
PARP: Poly ADP-ribose polymerase
PD-1/PD-L1: Programmed cell death protein 1/programmed cell death ligand 1
PI3K: phosphatidylinositol 3 kinase
PMN: Pre-metastatic niche
PTSG2: Prostaglandin-endoperoxide synthase 2
QX: Quxie capsule
ROS: Reactive oxygen species
RORγt: Retinoid-related orphan receptor-gammat
RP: RYP with Platycodon grandiflorum
RYP: Ruyiping
S6K1: Ribsosmal protein S6 kinase 1
SFI: ShenQi FuZheng injection
SGLX: Shugan Liangxue
Smad: Small mothers against decapentaplegic
SOCS3: Suppressor of cytokine signaling 3
STAT: Signal transducer and activator of transcription
T cell: T lymphocyte
TACE: Transcatheter arterial chemoembolization
TAMs: Tumor associated macrophages
TCM: Traditional Chinese medicine
TGF: Transforming growth factor
Th: Helper T lymphocytes
Th0: naïve T lymphocytes
THL: Tien-Hsien liquid
TIGIT: T-cell immunoreceptor with Ig and ITIM domains
Tim-3: T-cell immunoglobulin and mucin-domain containing-3
TME: Tumor microenvironment
TNF: Tumor necrosis factor
TNFR1: Tumor necrosis factor receptor superfamily member 1
Treg: Regulatory T cell
TSLP: Thymic stromal lymphopoietin
UCRCC: Ulcerative Colitis-Related Colorectal Cancer
VEGF: Vascular endothelial growth factor
XP: XIAOPI
XH: Xi Huang
YHD: Yanghe Decoction
YPF: Yu-ping-feng
YYFZBJS: Yi-Yi-Fu-Zi-Bai-Jiang-San
YYWY: Yangyin Wenyang
ZQT: Ze-Qi-Tang
